# Retrograde Intramedullary Nail Fixation for Derotational Femoral Osteotomy for Recurrent Femoropatellar Instability

**DOI:** 10.1155/2019/1893042

**Published:** 2019-09-11

**Authors:** Maximiliano Barahona, Alvaro Zamorano, Cristian Barrientos, Mauricio Guzmán, Yoshiro Sato, Jaime Hinzpeter

**Affiliations:** ^1^Orthopedic Department at Hospital Clinico Universidad de Chile, 999 Santos Dumont Street, Independencia, Santiago 8380456, Chile; ^2^Radiology Department at Hospital Clinico Universidad de Chile, 999 Santos Dumont Street, Independencia, Santiago 8380456, Chile

## Abstract

Axial alignment of the femur and tibia is often misdiagnosed in patients with patellofemoral stability problems. Femoral torsion is critical for patellofemoral biomechanics, so it must be evaluated in every patient before the plan of surgery is decided. This case describes a femoral derotational osteotomy due to excessive internal torsion of the femur fixed with a retrograde femoral nail. This type of fixation provides a biomechanical advantage compared to plates. At the two-year follow-up, the patient achieved excellent results, reaching a functional score of 91 points on the Lysholm scale. Derotational femoral osteotomy should be considered in patellofemoral pathology, and a retrograde femoral nail is a valid fixation method for this surgery.

## 1. Introduction

Patellofemoral instability is a multifactorial pathology, in which three main elements participate: lower limb malalignment, trochlear dysplasia, and the medial patellofemoral ligament (MPFL). Malalignment is a factor in a large percentage of patients who consult for this pathology, and the spectrum is broad. An increase in the distance between the intercondylar groove and the tibial tuberosity is just the tip of the iceberg; on the other end is the miserable malalignment syndrome, a rare condition that includes severe internal torsion of the femur [1, 2].

Femoral torsion is defined as the angle between the femoral neck axis and the condyle axis. The pathological cutoff value to perform a derotational osteotomy is a matter of current debate; one of the first values reported was those described by Cordier and Katthagen, who defined a normal range between 5° and 25° assuming a normal distribution of the data [3]. However, a range of two standard deviations from the mean sample does not imply a relation to a clinical problem like patella instability or pain.

Femoral torsion is a risk factor for patella instability [4]. A cadaveric study performed by Kaiser et al. [5] shows that a 10° increase of femoral torsion amplifies the lateral vector force to the patella, but a reconstruction of the MPFL alone can restore it; however, if the femoral torsion is now increased to 20°, the reconstruction of the MPFL alone is insufficient to decrease the lateral vector. This finding adds to the ever-growing evidence that supports the need to perform derotational osteotomies in patients with increased femoral torsion [6, 7].

Also, trochlea dysplasia may be a result of decreased contact between the femur and the patella due to an increased femoral torsion, emulating the proposed model in hip dysplasia [8]. So, when the trochlea angle is flat, femoral torsion must be evaluated and corrected if it is too high.

The knee surgeon must study the entire alignment in patella instability, including femoral and tibial torsion [9]. This case report is aimed at describing a femoral derotational osteotomy fixed by a retrograde femoral nail for recurrent patellar instability.

## 2. Case

The patient was a 17-year-old woman who complains of recurrent patellar instability in her left knee (5-7 episodes per year in the last three years) and in her right knee (two episodes). Physical examination revealed a full range of motion in both knees. The left knee had mild effusion and a positive apprehension test. On the right knee, no effusion or apprehension was noticed, but a pain in the medial retinaculum was found. The hip internal rotation was asymmetric, being greater in the left hip, so femoral malrotation was suspected. Tibial torsion, assessed by clinical thigh-foot angle, was normal. The preoperative patient-reported score was 19 on the Lysholm scale [10] and level 1 on the Tegner scale [11].

**Figure 1:**
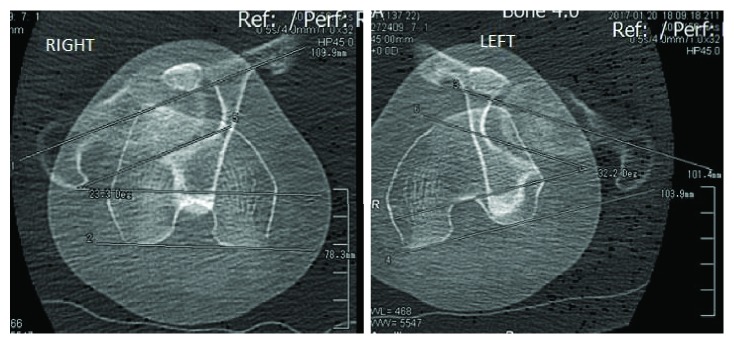
Preoperative CT scan femoral torsion measurements are shown. In the left leg, the femoral torsion is 32°; the right leg has a torsion of 23°. Measurements were made according to the technique described by Jarrett et al. [12].

All patients with recurrent patellar instability are studied with full-length standing anteroposterior (AP) radiography, computed tomography (CT) of the lower extremities, and knee magnetic resonance imaging (MRI) in our institution. CT measurements are summarised in Table 1, and an increased femoral internal rotation was the main finding (Figure 1). Femoral torsion was measured according to the technique described by Jarrett et al. [12], with an expected value of 15°. Radiography shows mild symmetric valgus alignment in the coronal plane, a femoral-tibial mechanical angle of 1° of valgus, and a mechanical distal lateral femoral angle (mDLFA) of 86° on the left knee. Knee MRI shows a bilateral lesion of the MPFL and chondral type 2 damage in the lateral facet of the left patella. The trochlea was flat on both knees, which means that it corresponds to a type B according to Dejour's classification [13].

According to the classification of patellar instability by Frosch and Schmeling [14], the right extremity was a type 2: instability without malalignment as femoral torsion was below 25°. The authors use the upper limit to indicate a femoral derotational osteotomy according to the findings of Kaiser et al. [5]; therefore, a medial patellofemoral ligament (MPFL) reconstruction alone was planned. The left extremity was a type 3e (instability with femoral malrotation); therefore, a femoral derotational osteotomy was planned.

Our planning was estimating the perimeter of the femur at the level of the osteotomy. For the estimation of the perimeter, in an axial section of the CT scan at the level of the desired osteotomy, we measure the radius in the anteroposterior (2,98 cm) direction and in the midlateral direction (2,66 cm). With these two measurements, we obtained an average radius of 2,82 cm. Then, the perimeter calculation is based on the formula 2∗*π*∗radius; therefore, the perimeter was 17,7 cm. The perimeter accounted for 360°, so for 17° of correction, a rotation of 0.8 cm should be made.

**Figure 2:**
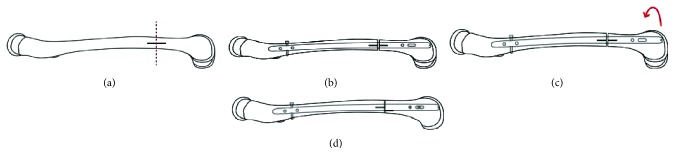
Illustrations that represent the surgery in the left femur. (a) The lateral cortex of the distal third of the femoral shaft was marked with a parallel line to the long axis to guide the correction (black line). A transverse bone cut (red line) parallel to the shaft axis was performed in the middle of the black line. (b) By a percutaneous approach to the knee, a retrograde femoral nail (T2, Stryker®) was placed. To begin with, a proximal blocking screw was placed in the dynamic hole. (c) The distal end of the osteotomy was externally rotated, as planned. (d) The parallel line to the axis of the diaphysis was the guide (black line), and the rotation was performed until both lines were separated by 0.8 cm. Immediately after the derotational maneuver, two distal locking screws were placed in the distal end of the nail.

Both knees were operated on during the same surgery. The first step was to perform the femoral osteotomy; for this, a lateral approach in the thigh was performed. The lateral cortex of the distal third of the femoral shaft was exposed. Two clamps were positioned on both sides of the desired location for the osteotomy, and the bone was marked parallel to the long axis. A transverse bone cut, parallel to the shaft axis, was performed in between the clamps and in the middle of the horizontal line with fluoroscopy guidance. Then, a retrograde femoral nail (T2, Stryker®) was used to fix the osteotomy. To begin, a proximal blocking screw was placed in the dynamic hole, then the distal end of the osteotomy was externally rotated, as planned, using the clamps to apply the force to achieve a distance of 0.8 cm between the horizontal lines at both ends of the osteotomy cut. Immediately after the derotational maneuver, two distal locking screws were placed in the distal end of the nail (Figure 2).

After the osteotomy was fixed, both the gracilis and the semitendinosus tendons of the left knee were harvested. The left MPFL was reconstructed with the gracilis autograft; it was fixed with two anchors in the patellar side and a biodegradable screw in the femur (BIORCI®, Smith & Nephew).

In the right knee, the MPFL was reconstructed with the semitendinosus of the contralateral knee using the same fixation method described for the left knee. In both knees, MPFL femoral attachments were selected using fluoroscopy as described by Schöttle et al. [15], and the grafts were fixed with 45° of knee flexion. In the left knee, while harvesting the hamstring tendon, an accidental partial lesion of the distal medial collateral ligament occurred and was repaired with an anchor.

After surgery, the patient did not present any complications. The rehabilitation protocol was weight bearing as tolerated, with two canes. Full knee extension was encouraged, and active flexion was limited to 60° for three weeks. After 10 weeks, she resumed walking without canes and gained full knee range of motion on the right knee and 100° in the left knee.

**Figure 3:**
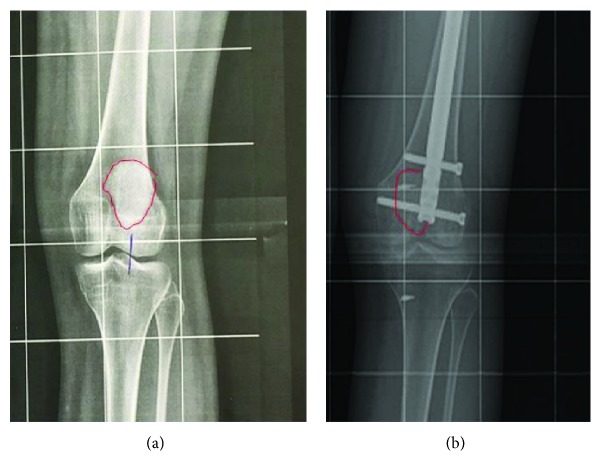
The enlarged image of the left knee of the full-length standing AP radiography before surgery (a) and enlarged image 8 months after surgery (b) are shown. Both were taken with 15° of internal rotation of the foot. The perimeter of the patella is highlighted in red, and the medialization of the patella due to the derotational osteotomy is observed.

**Figure 4:**
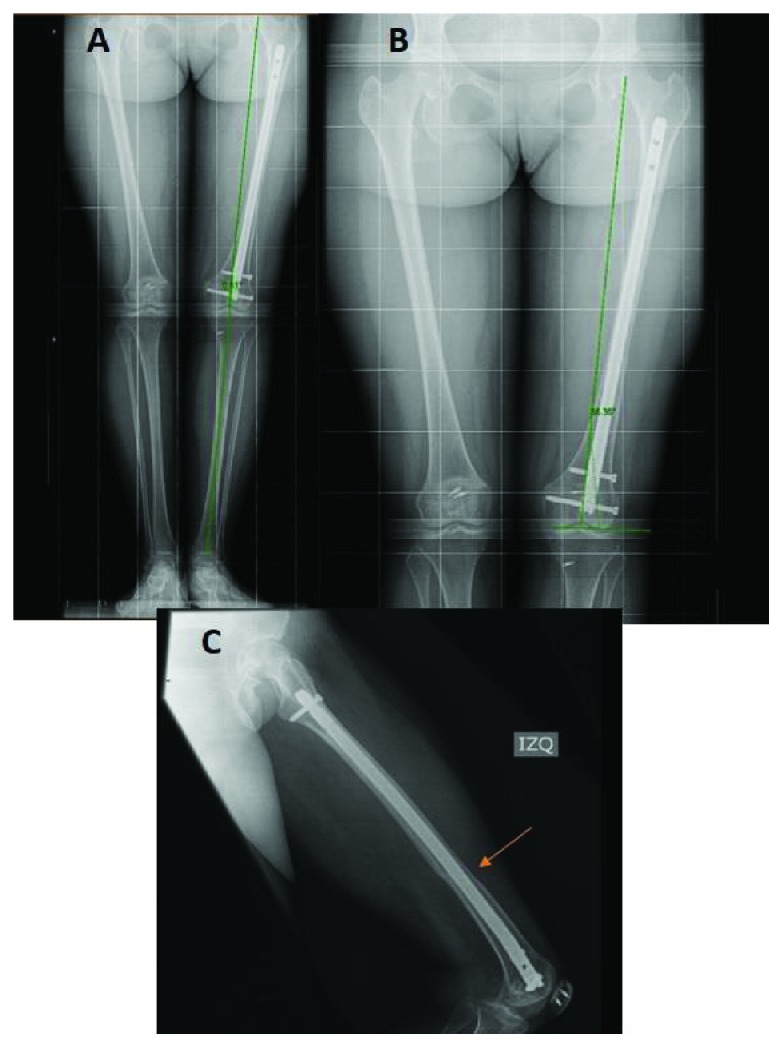
Radiographs at eight months after surgery are shown. (a) Full-length standing AP radiography of the lower extremity six months after surgery. Coronal alignment is symmetrical. (b) Femoral-tibial mechanical angle is 0.51° of valgus and mDLFA is 86°. (c) Lateral radiography of the femur. It shows complete healing of the osteotomy (red arrow). Also, the proximal locking of the nail and the entry point chosen in the distal femur for the LPFM can be observed.

**Figure 5:**
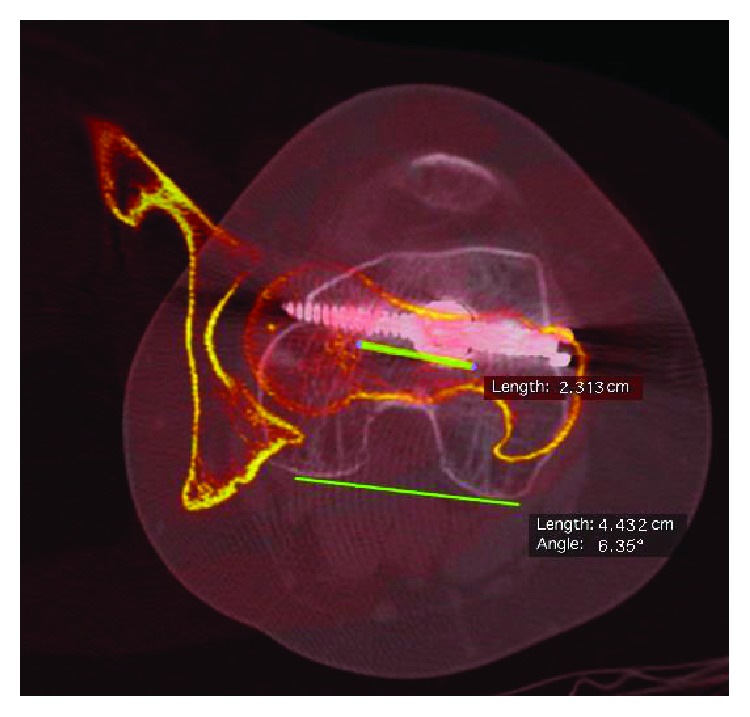
13-month postsurgery CT scan. The final femoral torsion that was achieved is shown, measuring 6.4°. Femoral torsion according to the method described by Jarrett et al. was used [12].

The eight-month postsurgery radiographs are shown in Figure 3 and are compared with the preoperative radiographs. It is remarkable how the patella looks medialised compared to the preoperative study (Figure 3), and as planned, the coronal alignment was not changed (Figure 4(a)) achieving a femoral-tibial mechanical angle after surgery of 0.51° of valgus. At the eight-month follow-up, the osteotomy was completely healed (Figures 4(b) and 4(c)). At the 18-month follow-up, a CT scan was performed, and femoral torsion was 6.4° (Figure 5). At the 24-month follow-up, the patient-reported outcome was 91 on the Lysholm scale [10] and level 4 on the Tegner scale [11]. The patient had no episodes of patella dislocation or instability complaints after the surgery.

## 3. Discussion

Axial alignment of the femur and tibia is often undervalued in patients with patellofemoral stability problems [9, 16]. Assessing femoral torsion is essential during the decision-making process for the surgical procedure to avoid poor surgical outcomes in patellofemoral pathologies [7]. Rotational alignment is crucial for knee biomechanics; knee arthroplasty is an excellent example of how patellar instability occurs when the femoral component is malrotated [17–20].

Recurrent patellofemoral instability is a multifactorial pathology; therefore, the evaluation should include the assessment of all factors that may be involved [21]. This must include MPFL, femoral torsion, tibial torsion, trochlea depth, patellar height, tibial tubercle to intercondylar groove distance (TT-TG), and coronal lower extremity alignment [2]. Femoral torsion plays an essential role in patellofemoral pathologies, so it must be evaluated in every patient before the surgery plan is defined for patellofemoral instability [2].

It is difficult to evaluate femoral torsion, both in the physical examination and in the radiological exams. In the former, an increase in passive internal hip rotation should raise a high suspicion for increased internal femoral torsion [22]. CT measurement is the gold standard to assess femoral torsion; however, MRI has been proposed, since it involves less radiation, despite having higher costs [23]. Also, some promissory findings have been reported with radiographs [24]. Another major problem lies in the lack of consensus on the anatomical landmarks chosen to perform the measurement. Kaiser et al. [25] showed that the six different methods of measurement proposed have a weak agreement. Our preference is to use the angle between the neck axis and the line that goes across both posterior condyles as proposed by Jarrett et al. [12].

The location in which to perform the derotational osteotomy in the femur is a matter of debate. According to findings of Seitlinger et al. [23], when patellar instability is due to excessive internal femoral torsion, the most critical factor is a lack of external rotation of the shaft of the femur which compensates for the normal or subtle excess of internal rotation of the femoral neck. Nevertheless, some studies have shown that the location of the derotational osteotomy in the femur may alter the coronal or sagittal alignment. Nelitz et al. [26] have proposed that if the coronal plane is in varus, the osteotomy must be performed in the distal metaphysis, and like in this case study, if the coronal alignment is neutral, a transverse osteotomy on the shaft of the femur must be done. However, Imhoff et al. demonstrated, in a cadaveric study, the importance of the orientation of the cut in the distal femur to avoid changes in the coronal orientation of the limb, when a distal femoral derotational osteotomy is performed [27]. More importantly, by a single cut in the distal femur, the coronal and the rotational problem of the patient can be addressed by choosing the patient-specific oblique cut [28]; these promising results must be validated in the operating room. In summary, the planning of the derotational osteotomy must consider the effects in the coronal and sagittal planes and the patient-specific segment of femur maltorsion. In this case, the distal shaft was chosen because the patient had normal coronal alignment, and according to Seitlinger et al., the problem must be the lack of external torsion of the shaft.

Few cases of femoral derotational osteotomy for patellofemoral instability are described in the literature; all of them are fixed with plates [29–33]. There are reports of successful cases of femoral derotational osteotomy, due to femoral fracture malunion, fixed with anterograde nails [34–36]. In this case report, a retrograde femoral nail was chosen for various reasons. First, biomechanically, the nail has a shorter lever arm than a lateral plate, making immediate weight bearing safer, and it can be allowed from day one after surgery. This was a significant factor, considering that both knees underwent surgery. Second, biomechanical studies have shown that nails have better torsion and axial stability compared to plates [37]. Bone healing after a femoral shaft fracture is 97% when fixed with a retrograde nail, and it is better when compared to plates and anterograde nails [38]. We believe that the same rate of bone healing can be expected in this type of osteotomy. Also, as the nail was first fixed in the proximal side and the distal end of the osteotomy was externally rotated, the presence of the nail inside the femur ensured that the fragment was rotated and not translated. In terms of cost, to use a plate or a nail is similar. The nail requires performing an arthrotomy; however, a minimum arthrotomy is also required for the MPFL reconstruction. Subtrochanteric fracture around the retrograde femoral nail is a well-known complication, and to leave the nail proximal to the lesser trochanter decreases the risk of this type of complication [39]. The nail's distal blocking screw placed 4 cm or lower from the knee articular line and blocking screws that radiographically extend to or beyond the medial cortex are more likely to cause pain and require removal, so it must be avoided [40]. In case of no union, it should be addressed in a similar way by using either a nail or a plate for fixation. Infection must be ruled out, and the need of bone grafting or additional mechanical stability should be evaluated [41]. Finally, if the patient requires a knee replacement in the future, since intramedullary guidance is needed to perform the cuts for the femoral component, both plate and nail must be removed. The nail can be removed by the same approach needed for the knee replacement; however, it requires an additional incision to remove the proximal blocking screw.

Femoral torsion correction was higher than planned; we aimed to correct at 17°, but instead, we corrected at 26°. According to our planning, instead of 0.8 cm, we made a rotation of 1.3 cm. Nevertheless, we still achieved a femoral torsion of 6.4° which is within our normal range of 5-25°, which is 10° lesser or higher [5] than the expected value of 15° according to the method described by Jarrett et al. [12]. To our knowledge, a precise method to achieve an excellent correlation between the correction planned and the correction obtained in derotational torsion has not been described. The accuracy of this method could be reported when we have more cases; however, we currently have not had a case outside 5°-25° of femoral torsion.

Derotational osteotomy can achieve a decrease in the load of the lateral patellofemoral compartment [8], as it was demonstrated in a cadaveric study by Liska et al. [42]. The decrease of the load in the compartment could at least avoid the progression of the chondral damage and even allow the cartilage to repair. This is also relevant to the case, in which the MRI showed ICRS chondral damage type 2 in the lateral facet of the left patella.

Postoperatory evolution was slower than expected; we think that the main reason was that both knees underwent surgery. After the 12-month follow-up, the patient showed excellent results and complete bone healing. Compared to the preoperative status, the patient now can ride a bike and has improved her performance in daily activities as Tegner and Lysholm scales show. Finally, the patient has not had any patellar dislocation or instability complain during the 24 months after surgery.

## 4. Conclusion

Femoral torsion has a crucial role in patellofemoral tracking; therefore, rotational alignment must always be assessed in recurrent patellofemoral instability. It may not be the most frequent finding, but if it is misdiagnosed, the treatment failure rate increases. Derotational femoral osteotomy is an excellent treatment option when internal femoral torsion is increased. The location on the femur where the osteotomy must be done ought to be chosen according to the segment of the femur that is more compromised and by the coronal and sagittal alignment of the femur. A retrograde femoral nail is a valid option for femoral derotational osteotomy fixation with biomechanical advantages compared to plates. The patient outcome after a two-year follow-up was excellent.

## Figures and Tables

**Table 1:** All CT measurements are shown. Femoral torsion was measured according to the method described by Jarrett et al. [12]. A femoral torsion of 32° was found in the left side, 17° higher than the expected value, so according to Kaiser et al. [5], a MPFL reconstruction alone is not enough to restore patellofemoral kinematics. The trochlea in both knees was type B according to the Dejour classification [13].

Measurement	Right	Left
Femoral torsion	**23.3°**	**32.2°**
Tibial torsion	**18°**	**21°**
Trochlea angle	**143°**	**147°**
TT-TG	**13 mm**	**14 mm**
Patellar height (Caton)	**0.98**	**0.87**
